# Risk factors of PSA progression and overall survival in patients with localized and locally advanced prostate cancer treated with primary androgen deprivation therapy

**DOI:** 10.1186/s12885-015-1429-0

**Published:** 2015-05-20

**Authors:** Atsushi Tomioka, Nobumichi Tanaka, Motokiyo Yoshikawa, Makito Miyake, Satoshi Anai, Yoshitomo Chihara, Eijiro Okajima, Akihide Hirayama, Yoshihiko Hirao, Kiyohide Fujimoto

**Affiliations:** Department of Urology, Nara Medical University, 840 Shijo-cho, Kashihara, Nara 634-8522 Japan

**Keywords:** Localized and locally advanced prostate cancer, Primary androgen deprivation therapy, Risk factors

## Abstract

**Background:**

Primary androgen deprivation therapy (PADT) has played an important role in the treatment of prostate cancer. We sought to identify factors of PSA progression in our series of patients with localized and locally advanced prostate cancer treated with PADT.

**Methods:**

Six-hundred forty-nine patients with localized and locally advanced prostate cancer who received PADT from 1998 to 2005 by Nara Uro-Oncology Research Group were enrolled. Age, T classification, stage, PSA level at diagnosis, Gleason score, laterality of cancer detected by biopsy and seminal vesicle involvement (SVI) were adopted as parameters of PSA progression. Cox’s proportional hazards model was used to determine the predictive factors for PSA progression.

**Results:**

The median follow-up period and the median PSA level at diagnosis were 49 months and 15 ng/mL. The 5-year disease specific survival rate, overall survival rate and PSA progression-free survival (PFS) rate were 97.9 %, 91.9 % and 71.2 %, respectively**.** The univariate analysis showed that the PSA level at diagnosis, Gleason score, laterality of cancer detected by biopsy and SVI were independent predictive parameters of PSA-PFS. However, by multivariate analysis, only laterality of cancer detected by biopsy (unilateral vs. bilateral) was an independent predictive parameter of PSA-PFS (p = 0.034). The patients were classified into new risk groups base on three factors: PSA level at diagnosis, Gleason score, and laterality of cancer detected by biopsy. The PSA-PFS rates at 5-years in the low- (none or one factor), intermediate- (two factors) and high-risk (three factors) groups were 78.2 %, 62.5 % and 46.9 % (p < 0.001), respectively.

**Conclusion:**

In localized or locally advanced prostate cancer patients who received PADT, laterality of cancer detected by biopsy was a significant predictor associated with a longer PSA-PFS. Our new risk grouping indicates the usefulness of PSA-PFS.

## Background

Androgen deprivation therapy (ADT) has played an important role in the treatment of prostate cancer since it was first reported by Huggins and Hodges [1]. Initially, ADT consisted of either surgical castration or estrogen administration. However, these procedures have problems related to the irreversible nature of castration and the side effects of estrogen administration on the cardiovascular system. These problems were resolved by suppression of the blood testosterone, which could be achieved by administration of a luteinizing hormone-releasing hormone (LH-RH) agonist, and the cardiovascular side-effects were reduced by anti-androgen agents. Combined androgen blockade (CAB) treatment using anti-androgen agents in combination with castration was developed, and was shown to facilitate stronger androgen suppression.

Widespread screening for prostate-specific antigen (PSA) has led to a significant increase in the detection of early stage, clinically localized prostate cancer. Currently, treatment of localized prostate cancer remains controversial. In the US it is frowned upon to give ADT for localized disease. The CaPSURE data from the USA indicated that 44 % of patients underwent radical prostatectomy, 23 % received definitive radiotherapy and 20 % received primary androgen deprivation therapy (PADT) [2]. On the other hand, the Japan Prostate Cancer Study Group showed the corresponding figures were 39.5 %, 23.9 % and 28.0 %, respectively [3] and the figures from Nara Uro-Oncological Research Group (NUORG) were 40 %, 16 % and 38 %, respectively [4–6]. As background of the present study, several reasons why Japanese patients with localized and locally advanced prostate cancer hesitate to undergo radical prostatectomy and prefer to receive PADT are proposed. Firstly, all patients are completely covered by the public health insurance system in Japan [5]. Secondly, Japanese patients tolerate hormonal therapy well without severe side effects for a long time [7, 8]. Thirdly, in those days radiotherapy was not widespread and doctors at hospitals where modalities for radiation therapy were not available usually chose PADT if the patients were unwilling to undergo radical prostatectomy [4, 5]. Fourthly, in those days, 49.9 % of the patients with localized or locally advanced prostate cancer were considered as the D’Amico high-risk group. 5-year biochemical recurrence-free rate in the D’Amico high-risk group treated with prostatectomy estimated 46.3 % [9]. 51 % of the patients with localized or locally advanced prostate cancer received PADT [4].

Recently, ADT is used as the primary treatment for advanced prostate cancer, and the efficacy of PADT for localized or locally advanced prostate cancer has also been reported [10, 11]. Mounting data on the efficacy and safety of ADT has brought about increased use of PADT in patients with localized or locally advanced prostate cancer in many countries, despite limited evidence to date on the impact on clinical outcomes [12–14].

We performed a retrospective study of the efficacy of PADT and identified risk factors for PSA progression in our series of patients with localized and locally advanced prostate cancer.

## Methods

This study retrospectively evaluated 649 Japanese patients with localized and locally advanced prostate cancer who received PADT following diagnosis by the NUORG between January 1998 and December 2005. The diagnosis was based on prostate biopsy. Computed tomography, bone scans, magnetic resonance imaging and/or transrectal ultrasonography were used in all cases. These patients selected PADT for various reasons, including older age, patient’s preference and comorbidity such as severe cardiovascular disease or other malignancies, although definitive therapy such as radical prostatectomy or irradiation is the standard treatment for patients with localized prostate cancer.

Follow-up data were retrieved from hospital medical records. Patients were followed every month for the first 3 months and every 3 months thereafter. PSA progression was defined as the first day when the PSA was increased for three consecutive times or when clear clinical radiological evidence of progressive disease was seen. PSA progression-free survival (PFS) rate was estimated by the Kaplan-Meier method and the log rank test was used to assess differences between groups: Age (≤75 vs. 76≤), T classification, stage (B vs. C), PSA level at diagnosis (<10 ng/mL vs. 10–20 ng/mL vs. 20 ng/mL≤), Gleason score (6 vs. 7 vs. 8≤), laterality of cancer detected by biopsy (unilateral vs. bilateral) and seminal vesicle involvement (SVI; negative vs. positive). Based on the result of the log rank test, the Cox proportional hazards regression model was performed to analyze independent predictors of PSA progression.

We classified the patients into the modified D’Amico risk groups [15] and the Japan Cancer of the prostate Risk Assessment (J-CAPRA) risk groups [16]. The modified D’Amico risk grouping classifies patients into three risk groups based on PSA level at diagnosis and Gleason score: low- (PSA level at diagnosis ≤10 ng/mL and Gleason score ≤ 6; 112 patients), intermediate- (10 ng/mL < PSA level at diagnosis ≤20 ng/mL and/or Gleason score 7; 203 patients), and high- risk (PSA level at diagnosis >20 ng/mL or 8 ≤ Gleason score; 334 patients). In J-CAPRA risk grouping, patients were assigned 1 point for Gleason score 7 and 2 points for Gleason score 8 to 10; 1 point for PSA level at diagnosis 20 to 100 ng/mL, 2 points for PSA 100 to 500 ng/mL, and 3 points for PSA higher than 500 ng/mL; 1 point for stage T2c or T3a, 2 points for T3b, and 3 points for T4. Points for each variable are summed to yield a total score with a range of 0 to 12. The J-CAPRA score was also categorized to identify three groups at low- (0 to 2 points; 459 patients), intermediate- (3 to 7 points; 190 patients) and high- (8 to 12 points; 0 patient) risk of recurrence.

Statistical analysis was performed SPSS 11.0 J (SPSS Inc., Chicago, Illinois) and p < 0.05 was considered statistically significant. The product limit method of Kaplan-Meier was used to assess survival. The log-rank method was used to assess differences between groups. The Cox proportional hazards model was performed to analyze independent predictors of PSA-PFS. Only the variables that were found to be significant in the univariate analyses (p < 0.05) were entered into the multivariate analysis to determine the most significant factor for predicting disease outcome.

The Medical Ethics Committee of Nara Medical University approved this retrospective study.

## Results

**Fig. 1:**
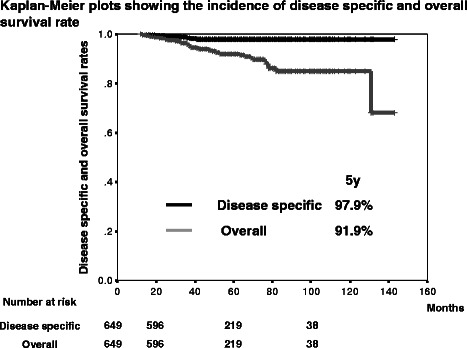
Kaplan-Meier plots showing the incidence of disease specific and overall survival rate

**Fig. 2:**
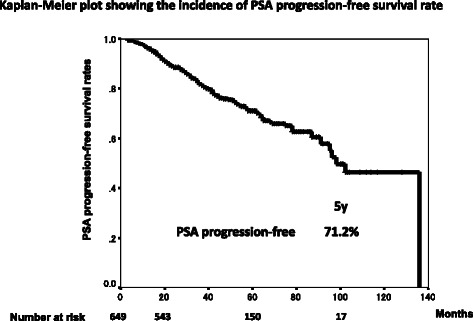
Kaplan-Meier plot showing the incidence of PSA progression-free survival rate

**Fig. 3:**
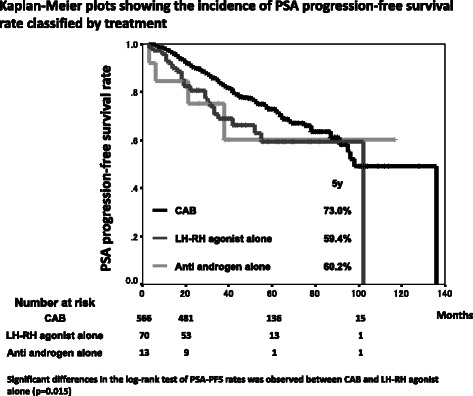
Kaplan-Meier plots showing the incidence of PSA progression-free survival rate classified by treatment

The median age, the median follow-up period and the median PSA level at diagnosis were 77 years (mean: 76.5; range: 53–95), 49 months (mean: 52.0; range: 12–143) and 15 ng/mL (mean: 28.9; range: 1.4 – 200), respectively (Table 1). The number of patient cores was varied and the median was 7 (mean: 8.03; range: 2–25). The 5-year overall survival rate was 91.9 % and 7.6 % of patients (49 patients) died during follow-up. The 5-year disease specific survival rate was 97.9 % and 1.2 % of patients (8 patients) died of prostate cancer (Fig. 1). The 5-year PSA-PFS rates were 71.2 % (Fig. 2). A total of 566 patients (87.2 %), 70 patients (10.8 %) and 13 patients (2.0 %) were treated with CAB, LH-RH agonist alone and anti-androgen alone, respectively. Significant differences in a log-rank test of PSA-PFS rates was observed between CAB and LH-RH agonist alone (p = 0.015) (Fig. 3).

**Table 1:** Characteristic of patients

	Median	Mean	Range
Age (years)	77	76.5	53-95
Follow-up period (months)	49	52.0	12-143
PSA level at diagnosis (ng/mL)	15	28.9	1.4-200

Table 2 shows the results of analysis for PSA-PFS by a log-rank test. T classification (T1c vs.T2c; p = 0.036, T1c vs.T3b; p = 0.028, T2ab vs.T2c; p = 0.001, T2ab vs. T3b; p = 0.003), PSA level at diagnosis (10–20 ng/mL vs. 20 ng/mL≤; p = 0.002, <10 ng/mL vs. 20 ng/mL≤; p < 0.001), Gleason score (7 vs. 8≤; p = 0.028, 6 vs. 8≤; p = 0.004), laterality of cancer detected by biopsy (unilateral vs. bilateral; p < 0.001) and SVI (negative vs. positive; p = 0.030) were significantly associated with PSA-PFS.

**Table 2:** Results of analysis of PSA-PFS by log-rank test

		No. of patients	No. of PSA progression	5 year PSA-PFS rate	log-Rank
Total		649	161	71.2 %	
Age	≤75	265	66	73.9 %	
	76≤	384	95	69.0 %	0.230
T classification	T1c	189	42	71.8 %	
	T2ab	205	37	78.9 %	
	T2c	104	36	65.2 %	*
	T3a	103	28	70.1 %	
	T3b	48	18	54.0 %	
Stage	B	498	115	73.2 %	
	C	151	46	64.9 %	0.155
PSA level at diagnosis	<10	197	31	83.2 %	
	10-20	195	42	75.2 %	**
	20≤	257	88	59.4 %	
Gleason score	6	288	60	76.2 %	
	7	196	43	72.0 %	***
	8≤	165	58	61.8 %	
Laterality of cancer detected by biopsy	Unilateral	365	66	77.2 %	
	Bilateral	284	95	63.8 %	<0.001
SVI	Negative	601	143	72.7 %	
	Positive	48	18	54.0 %	0.030

PSA-PFS: PSA progression-free survival

SVI: seminal vesicle involvement

^*^: T1c vs. T2c; *p* = 0.036. T1c vs. T3b; *p* = 0.028. T2ab vs. T2c; *p* = 0.001. T2ab vs. T3b; *p* = 0.003. others; *p* > 0.05

**: <10 vs. 10–20; *p* = 0.192. 10–20 vs. 20≤; *p* = 0.002. <10 vs. 20≤; *p* < 0.001

***: 6 vs. 7, *p* = 0.310. 7 vs. 8≤; *p* = 0.028. 6 vs. 8≤; *p* = 0.004

We used a Cox’s proportional hazards model to determine the predictive parameter of PSA progression. Based on the result of the log-rank test, age (≤75 vs. 76≤), stage (B vs. C), PSA level at diagnosis (<20 ng/mL vs. 20 ng/mL≤), Gleason score (≤7 vs. 8≤), laterality of cancer detected by biopsy (unilateral vs. bilateral) and SVI (negative vs. positive) were adopted as clinicopathological parameters of PSA progression and T classification was excluded as a parameter in grouping the patients, because it was difficult to distinguish the cutoff point. PSA level at diagnosis, Gleason score, laterality of cancer detected by biopsy and SVI were the significant factors for a longer PSA-PFS. But, by multivariate analysis, only laterality of cancer detected by biopsy was an independent predictive parameter of PSA-PFS (Hazard ratio: 1.523, p = 0.034, 95 % confidence interval: 1.033-2.245) (Table 3).

**Table 3:** Results of analysis of PSA-PFS by Cox proportional hazards model

			Univariate			Multivariate	
		Hazard-ratio	*p*	95 % CI	Hazard-ratio	*p*	95 % CI
Age	≤75	1					
	76≤	1.213	0.232	0.884- 1.666			
Stage	B	1					
	C	1.283	0.157	0.908-1.813			
PSA level at diagnosis	<20	1			1		
	20≤	2.048	<0.001	1.500-2.797	1.613	0.071	0.959-2.711
Gleason score	≤7	1			1		
	8≤	1.734	0.001	1.256-2.393	1.436	0.064	0.979-2.107
Laterality of cancer detected by biopsy	unilateral	1			1		
	bilateral	2.102	<0.001	1.533- 2.883	1.523	0.034	1.033-2.245
SVI	Negative	1			1		
	Positive	1.71	0.032	1.046- 2.793	1.109	0.753	0.582-2.113

PSA-PFS: PSA progression-free survival

95 % CI: 95 % confidence interval

SVI: seminal vesicle involvement

**Fig. 4:**
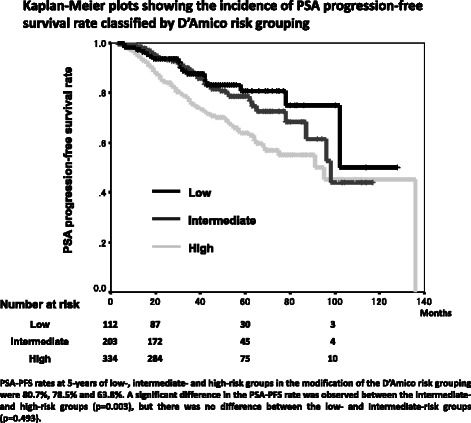
Kaplan-Meier plots showing the incidence of PSA progression-free survival rate classified by D’Amico risk grouping

**Fig. 5:**
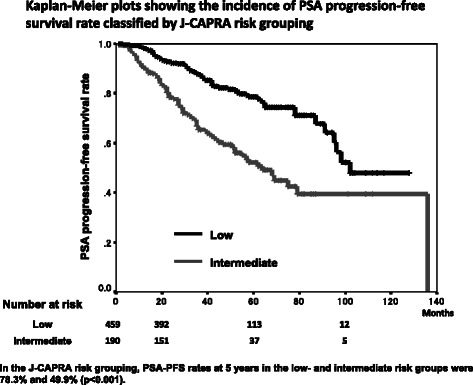
Kaplan-Meier plots showing the incidence of PSA progression-free survival rate classified by J-CAPRA risk grouping

PSA-PFS rates at 5 years in low-, intermediate- and high-risk groups by the modified D’Amico risk grouping were 80.7 %, 78.5 % and 63.8 % (Fig. 4). A significant difference in PSA-PFS rate was observed between the intermediate- and high-risk groups (p = 0.003), but there was no difference between the low- and intermediate-risk groups (p = 0.493), as reported by Ueno *et al.* [14]. In the J-CAPRA risk grouping, PSA-PFS rates at 5 years in the low- and intermediate- risk groups were 78.3 % and 49.9 % (p < 0.001) (Fig. 5).

**Fig. 6:**
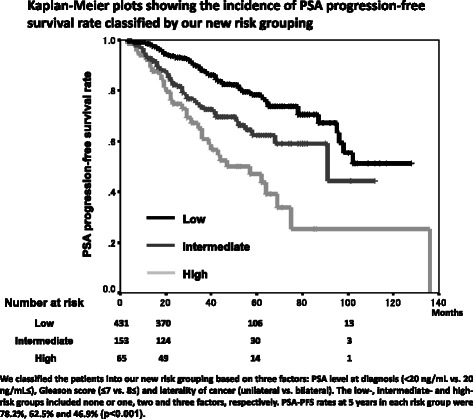
Kaplan-Meier plots showing the incidence of PSA progression-free survival rate classified by our new risk grouping

We classified the patients into our new risk groups based on three factors: PSA level at diagnosis (<20 ng/mL vs. 20 ng/mL≤), Gleason score (≤7 vs. 8≤) and laterality of cancer detected by biopsy (unilateral vs. bilateral). The low- (431 patients), intermediate- (153 patients) and high-risk (65 patients) groups included none or one, two and three factors, respectively. PSA-PFS rates at 5 years in the respective risk groups were 78.2 %, 62.5 % and 46.9 %, respectively, and a significant difference in the PSA-PFS rate was observed between groups by the log-rank test (p < 0.001) (Fig. 6). The Cox proportional hazards model showed the same result as the log-rank test (Table 4).

**Table 4:** The Cox proportional hazards model of PSA-PFS

		Hazard-ratio	*p*	95 % CI
D’Amico	Low	1		
	Intermediate	1.212	0.493	0.693-2.119
	High	2.116	0.003	1.289-3.492
J-CAPRA	Low	1		
	Intermediate	2.623	<0.001	1.942-3.542
Our new grouping	Low	1		
	Intermediate	1.936	<0.001	1.350-2.778
	High	3.408	<0.001	2.269-5.117

PSA-PFS: PSA progression-free survival

95 % CI: 95 % confidence interval

## Discussion

Although PADT has been widely used for the treatment of prostate cancer at any early disease stage, there is not much information regarding the clinical outcomes associated with clinically localized and locally advanced prostate cancer treated by PADT. According to some reports, a survival advantage of CAB over castration monotherapy has been indicated [10, 16, 17]. Thus, the focus of the present study was placed on CAB rather than castration monotherapy as PADT to evaluate its efficacy in terms of long-term disease control of clinically localized and locally advanced prostate cancer.

At the present time, the younger patients with localized prostate cancer and locally advanced prostate cancer without complications have a tendency to select the radical treatment such as prostatectomy. But, in those days, the use of PADT was still common in patients with localized prostate cancer and locally advanced prostate cancer in Japan [4, 6]. In many cases, the patients might select PADT by older age or some complications.

In this study, the PSA-PFS (71.2 % at 5 years) was similar to other previous reports [10, 16–18]. These results were worse than other treatment modalities such as prostatectomy and radiotherapy. If the PSA progression was defined as the day when the PSA at least 4 weeks later was 25 % increase over nadir with more than 2 ng/mL, the PSA-PFS might be better. The disease specific survival rate was very high (97.9 % at 5 years) even though 23.3 % of patients had stage C, suggesting a possible cure of localized and locally advanced prostate cancer by PADT. Egawa *et al*. reported that PADT was as effective as radical prostatectomy with regard to disease-specific survival rate in localized prostate cancer [19] and Akaza *et al.* demonstrated no difference in overall survival in patients with localized prostate cancer treated with PADT and men of the same age among the general population, suggesting that there is no increase in the mortality of men treated with PADT [7].

Although prospective data defining the risks and benefits are lacking, clinical practice trends show an increased use of ADT as monotherapy. Date from the results of the CAPSURE™ survey shows an increase in the incidence of patients treated with PADT from 1989 to 2000, during which time the percent assigned to the low-, intermediate- and high-risk groups increased from 4.6 % to 14.2 %, 8.9 % to 19.7 % and 32.8 % to 48.2 %, respectively [20].

On the other hand, there is growing evidence that ADT is associated with an increased risk of various comorbidities including ischemic heart disease, metabolic syndrome, glucose intolerance, and a decrease in bone mineral density [21–24]. As a result, patients who received PADT have worse overall survival compared with conservative management [25, 26]. In contrast, several reports have also shown no significant increase in cardiovascular mortality with ADT in men with prostate cancer [27–29].

Several parameters were isolated as predictors of PSA progression. Nadir PSA level and the percentage of positive biopsy cores remained as independent prognostic factors on multivariate analysis [18]. Younger patients (<70 years) and those with 6 ≤ Gleason score were at a higher risk of treatment failure [30]. Ueno *et al.* reported that PSA ≤20 ng/mL, Gleason score ≤7, and time to nadir PSA ≤6 months showed a good response to PADT [17]. In this study, there was no difference between Gleason score of 6 vs. 7 ≤ (p = 0.310), and we adopted a Gleason score ≤7 vs. 8 ≤ as a parameter of PSA progression in Cox’s proportional hazards model. We found that PSA level at diagnosis, Gleason score, laterality of cancer detected by biopsy and SVI were significant factors for a longer PSA-PFS, except for age and stage by univariate analysis. Then, by multivariate analysis, only laterality of the cancer detected by biopsy was an independent predictive parameter of PSA-PFS.

Firstly, we classified the patients using our four new risk groups: no, one, two and three factors. No significance difference was shown between the no factor (34.2 %, 222 of 649 patients) and one factor (32.2 %, 209 of 649 patients) (p = 0.091). Therefore we combined the no and one factor into the low-risk group.

Our low-risk factor patients accounted for two-thirds of the T1c-T3b patients. For patients showing a good response to ADT, ADT showed an excellent effect in this study. This effect may be explained by the observation that resected specimens after neoadjuvant ADT were sometimes completely apoptotic. Kitagawa *et al*. analyzed the histological effects of ADT in specimens from patients treated with radical prostatectomy after neoadjuvant ADT [31]. They reported that histologically cured or nearly cured patients accounted for more than 40 % of the total number. In addition, the recurrence-free survival rate of patients with complete apoptosis was 100 %. These results supported our observation that some cases of localized prostate cancer could be cured by ADT alone. Schulman *et al.* also performed neoadjuvant ADT for 3 months before radical prostatectomy in patients with localized prostate cancer, and good histopathological effects [32].

In the modified D’Amico risk grouping, a significant difference in PSA-PFS rate was observed between the intermediate- and high-risk groups, but there was no difference between the low- and intermediate-risk groups. The J-CAPRA risk grouping included also metastatic cancer patients in addition to localized and locally advanced prostate cancer. Our new risk groups included only localized and locally advanced prostate cancer patients and a significant difference of PSA-PFS rate was observed between all groups. Our new risk grouping indicates the usefulness for localized and locally advanced prostate cancer patients treated with PADT.

There are several limitations to the current study. Firstly, there may be interobserver variation of the Gleason score between general pathologists and uropathologists. Secondly, the current study is retrospective and results should be interpreted accordingly.

## Conclusions

Unilateral positive biopsy was a significant predictor associated with a longer PSA-PFS in localized or locally advanced prostate cancer patients who received PADT. Our new risk groups according to the three factors of PSA level at diagnosis, Gleason score and laterality of cancer detected by biopsy indicate the usefulness for PSA-PFS. The efficacy and toxicity of ADT for localized or locally advanced prostate cancer requires further study before it can be recommended as the primary treatment. In the future, a prospective randomized study or comparative study of QOL or medical cost compared with other treatments will be necessary to establish PADT as a recommended treatment for early prostate cancer. Our results provide potentially clinical useful predictive tools for physicians and patients contemplating PADT for localized or locally advanced prostate cancer as well as the outcomes necessary to design prospective studies of the treatment strategy.

## Abbreviations

ADT: Androgen deprivation therapy
LH-RH: Luteinizing hormone-releasing hormone
CAB: Combined androgen blockade
PSA: Prostate specific antigen
PADT: Primary androgen deprivation therapy
NUORG: Nara Uro-Oncological Research Group
PFS: Progression-free survival
SVI: Seminal vesicle involvement

## References

[1] Huggins C, Hodges CV (1972). Studies on prostatic cancer. I. The effect of castration, of estrogen and androgen injection on serum phosphatases in metastatic carcinoma of the prostate. CA Cancer J Clin.

[2] Cooperberg MR, Lubeck DP, Penson DF, Mehta SS, Carroll PR, Kane CJ (2003). Sociodemographic and clinical risk characteristics of patients with prostate cancer within the Veterans Affairs health care system: data from CaPSURE. J Urol.

[3] Onozawa M, Hinotsu S, Tsukamoto T, Oya M, Ogawa O, Kitamura T (2014). Recent trends in the initial therapy for newly diagnosed prostate cancer in Japan. Jpn J Clin Oncol.

[4] Tanaka N, Fujimoto K, Hirayama A, Yoneda T, Yoshida K, Hirao Y (2010). Trends of the primary therapy for patients with prostate cancer in Nara uro-oncological research group (NUORG): a comparison between the CaPSURE data and the NUORG data. Jpn J Clin Oncol.

[5] Tanaka N, Fujimoto K, Hirayama A, Samma S, Momose H, Kaneko Y (2011). The primary therapy chosen for patients with localzed prostate cancer between the university hospital and its affiliated hospital in Nara Uro-Oncological Research Group redistration. BMC Urol.

[6] Tanaka N, Hirayama A, Yoneda T, Yoshida K, Konishi N, Fujimoto K (2013). Trends of risk classification and primary therapy for Japanese patients with prostate cancer in Nara uro-oncological research group (NUORG): a comparison between 2004–2006 and 2007–2009. BMC Cancer.

[7] Akaza H, Homma Y, Usami M, Hirao Y, Tsushima T, Okada K (2006). Efficacy of primary hormone therapy for localized or locally advanced prostate cancer: results of a 10-year follow-up. BJU Int.

[8] Akaza H (2010). Future prospects for luteinizing hormone-releasing hormone analogues in prostate cancer treatment. Pharmacology.

[9] Tanaka N, Fujimoto K, Hirayama A, Torimoto K, Okajima E, Tanaka M (2011). Risk-strarified survival rates and predictors of biochemical recurrence after radical prostatectomy in Nara, Japan, cohort study. Int J Clin Oncol.

[10] Akaza H, Homma Y, Okada K, Yokoyama M, Usami M, Hirao Y (2003). A prospective and randomized study of primary hormonal therapy for patients with localized or locally advanced prostate cancer unsuitable for radical prostatectomy: results of the 5-year follow-up. BJU Int.

[11] Labrie F, Candas B, Gomez JL, Cusan L (2002). Can combined androgen blockade provide long-term control or possible cure of localized prostate cancer?. Urology.

[12] Cooperberg MR, Grossfeld GD, Lubeck DP, Carroll PR (2003). National practice patterns and time trends in androgen ablation for localized prostate cancer. J Natl Cancer Inst.

[13] Shahinian VB, Kuo YF, Freeman JL, Orihuela E, Goodwin JS (2005). Increasing use of gonadotropin-releasing hormone agonists for the treatment of localized prostate carcinoma. Cancer.

[14] Weight CJ, Klein EA, Jones JS (2008). Androgen deprivation falls as orchiectomy rates rise after changes in reimbursement in the U.S. Medicare population. Cancer.

[15] D'Amico AV, Whittington R, Malkowicz SB, Schultz D, Blank K, Broderick GA (1998). Biochemical outcome after radical prostatectomy, external beam radiation therapy, or interstitial radiation therapy for clinically localized prostate cancer. JAMA.

[16] Hinotsu S, Akaza H, Usami M, Ogawa O, Kagawa S, Kitamura T (2007). Current status of endocrine therapy for prostate cancer in Japan analysis of primary androgen deprivation therapy on the basis of data collected by J-CaP. Jpn J Clin Oncol.

[17] Ueno S, Namiki M, Fukagai T, Ehara H, Usami M, Akaza H (2006). Efficacy of primary hormonal therapy for patients with localized and locally advanced prostate cancer: a retrospective multicenter study. Int J Urol.

[18] Kobayashi M, Nukui A, Suzuki K, Kurokawa S, Morita T. Clinical efficacy of primary combined androgen blockade for Japanese men with clinically localized prostate cancer unsuitable for local definitive treatment: a single institution experience. Int J Clin Oncol. 2011;16(6):630-6. 10.1007/s10147-011-0232-4 21512893

[19] Egawa M, Misaki T, Imao T, Yokoyama O, Fuse H, Suzuki K (2004). Retrospective study on stage B prostate cancer in the Hokuriku District. Japan Int J Urol.

[20] Cancer Registrarion Committee of Japan Urological Association (2005). Clinicopathological statistics on registered prostate cancer patients in Japan: 2000 report from the Japanese Urological Association. Int J Urol.

[21] Braga-Basaria M, Dobs AS, Muller DC, Carducci MA, John M, Egan J (2006). Metabolic syndrome in men with prostate cancer undergoing long-term androgen-deprivation therapy. J Clin Oncol.

[22] D'Amico AV, Denham JW, Crook J, Chen MH, Goldhaber SZ, Lamb DS (2007). Influence of androgen suppression therapy for prostate cancer on the frequency and timing of fatal myocardial infarctions. J Clin Oncol.

[23] Keating NL, O'Malley AJ, Freedland SJ, Smith MR (2010). Diabetes and cardiovascular disease during androgen deprivation therapy: observational study of veterans with prostate cancer. J Natl Cancer Inst.

[24] Shahinian VB, Kuo YF, Freeman JL, Goodwin JS (2005). Risk of fracture after androgen deprivation for prostate cancer. N Engl J Med.

[25] Lu-Yao GL, Albertsen PC, Moore DF, Shih W, Lin Y, DiPaola RS (2008). Survival following primary androgen deprivation therapy among men with localized prostate cancer. JAMA.

[26] Wong YN, Freedland S, Egleston B, Hudes G, Schwartz JS, Armstrong K (2009). Role of androgen deprivation therapy for node-positive prostate cancer. J Clin Oncol.

[27] Efstathiou JA, Bae K, Shipley WU, Hanks GE, Pilepich MV, Sandler HM (2009). Cardiovascular mortality after androgen deprivation therapy for locally advanced prostate cancer: RTOG 85–31. J Clin Oncol.

[28] Roach M, Bae K, Speight J, Wolkov HB, Rubin P, Lee RJ (2008). Short-term neoadjuvant androgen deprivation therapy and external-beam radiotherapy for locally advanced prostate cancer: long-term results of RTOG 8610. J Clin Oncol.

[29] Studer UE, Whelan P, Albrecht W, Casselman J, de Reijke T, Hauri D (2006). Immediate or deferred androgen deprivation for patients with prostate cancer not suitable for local treatment with curative intent: European Organisation for Research and Treatment of Cancer (EORTC) Trial 30891. J Clin Oncol.

[30] Janoff DM, Peterson C, Mongoue-Tchokote S, Peters L, Beer TM, Wersinger EM (2005). Clinical outcomes of androgen deprivation as the sole therapy for localized and locally advanced prostate cancer. BJU Int.

[31] Kitagawa Y, Koshida K, Mizokami A, Komatsu K, Nakashima S, Misaki T (2003). Pathological effects of neoadjuvant hormonal therapy help predict progression of prostate cancer after radical prostatectomy. Int J Urol.

[32] Schulman CC, Debruyne FM, Forster G, Selvaggi FP, Zlotta AR, Witjes WP (2000). 4-Year follow-up results of a European prospective randomized study on neoadjuvant hormonal therapy prior to radical prostatectomy in T2-3N0M0 prostate cancer. European Study Group on Neoadjuvant Treatment of Prostate Cancer. Eur Urol.

